# Correction: Serum-IgG responses to SARS-CoV-2 after mild and severe COVID-19 infection and analysis of IgG non-responders

**DOI:** 10.1371/journal.pone.0258401

**Published:** 2021-10-04

**Authors:** Emelie Marklund, Susannah Leach, Hannes Axelsson, Kristina Nyström, Heléne Norder, Mats Bemark, Davide Angeletti, Anna Lundgren, Staffan Nilsson, Lars-Magnus Andersson, Aylin Yilmaz, Magnus Lindh, Jan-Åke Liljeqvist, Magnus Gisslén

Fig 3 is incorrect. The chart A.IgG mislabels columns IgG-, M, and S. The authors have provided a corrected version here.

**Fig 3:**
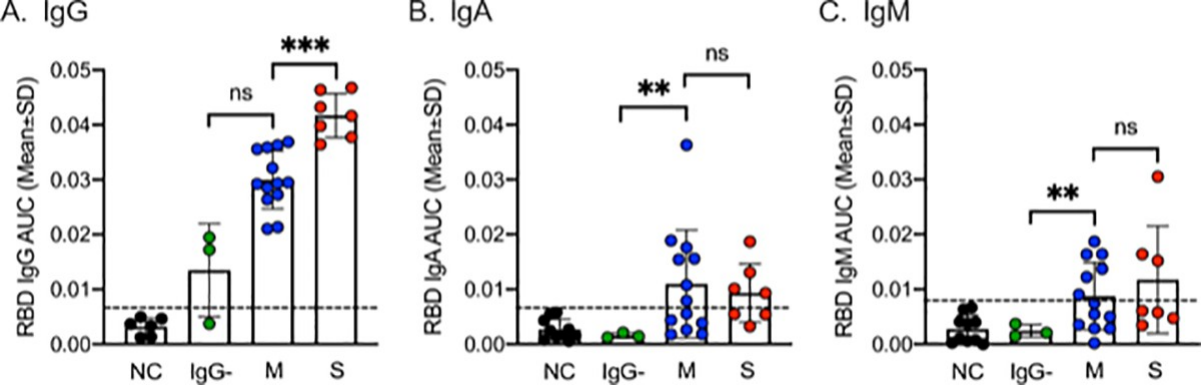
Antibody responses against the receptor-binding domain of SARS-CoV-2. RBD-specific serum IgG (A), IgA (B) and IgM (C) antibodies in patients with severe symptoms (red, n = 7), mild symptoms and IgG-positive (blue, n = 13), mild symptoms and IgG-negative (green, n = 3) collected 78–91 days post symptom onset. NC = negative controls (black dots, n = 10). Cut-off (mean + 2SD of NC AUC) indicated by dashed lines. ns P>0.05, * P<0.05, ** P<0.01.
